# Machine learning denoising of high-resolution X-ray nano­tomography data

**DOI:** 10.1107/S1600577521011139

**Published:** 2022-01-01

**Authors:** Silja Flenner, Stefan Bruns, Elena Longo, Andrew J. Parnell, Kilian E. Stockhausen, Martin Müller, Imke Greving

**Affiliations:** a Helmholtz-Zentrum Hereon, Max-Planck-Strasse 1, 21502 Geesthacht, Germany; b Department of Physics and Astronomy, University of Sheffield, Western Bank, Sheffield S3 7RH, United Kingdom; c Department of Osteology and Biomechanics, University Medical Center, Lottestrasse 55a, 22529 Hamburg, Germany

**Keywords:** nanotomography, full-field X-ray microscopy, Zernike phase contrast, machine learning, denoising

## Abstract

High-resolution X-ray nano­tomography is a quantitative tool for investigating specimens from a wide range of research areas. However, the quality of the reconstructed tomogram is often obscured by noise and therefore not suitable for automatic segmentation. Filtering methods are often required for a detailed quantitative analysis. However, most filters induce blurring in the reconstructed tomograms. Here, machine learning (ML) techniques offer a powerful alternative to conventional filtering methods. In this article, we verify that a self-supervised denoising ML technique can be used in a very efficient way for eliminating noise from nano­tomography data. The technique presented is applied to high-resolution nano­tomography data and compared to conventional filters, such as a median filter and a nonlocal means filter, optimized for tomographic data sets. The ML approach proves to be a very powerful tool that outperforms conventional filters by eliminating noise without blurring relevant structural features, thus enabling efficient quantitative analysis in different scientific fields.

## Abstract 2

A high-performance denoising filter based on machine learning for high-resolution synchrotron nano­tomography data is analyzed and evaluated.

## 1. Introduction

Hard X-ray nano­tomography is a commonly used tool in many research areas, such as materials science, biology and medicine. Transmission X-ray microscopes (TXM) are often equipped with diffractive optics [beam-shaping condenser (Jefimovs *et al.*, 2008 ▸) and Fresnel zone plates (FZP)], where, in particular at higher X-ray energies, the efficiency is very low, resulting in a low signal-to-noise ratio (SNR) at the detector plane. Capillary optics provide higher flux but are more difficult to combine with phase con­trast methods such as Zernike phase con­trast (Zernike, 1942 ▸; Schmahl *et al.*, 1994 ▸) as they lack sensitivity. Recent developments have led to a significant increase in time resolution, in particular at synchrotron-based nano­tomography setups. Full-field TXM setups have reached scan times in the minute regime (Ge *et al.*, 2018 ▸) and even scan times of down to 6 s have been reported recently (Flenner *et al.*, 2020 ▸). This allows not only a push for *in situ* nano­tomography experiments at high temporal resolution but also the chance to reduce the dose for radiation-sensitive samples such as biological or medical specimens.

While nano­tomography offers high spatial resolution, there is one key challenge: even small sample movements at the nanoscale become visible and reduce the image quality. In addition, the dose on the sample is generally not negligible, due to the focusing of the beam by the beam shaper onto the sample and the resulting increased flux density. In particular for biological samples, this can lead to structural changes during measurement (*e.g.* shrinking or bubbling), resulting in severe artifacts in the tomographic reconstruction. One strategy to minimize the impact of the dose on the sample is to minimize the scan time as much as possible. *In situ* experiments are another example where fast scan times are crucial: the scan time has to be significantly shorter than the dynamic process observed to prevent movement artifacts in the tomograms. For both examples, however, there is always a trade-off between short scan times and a significant increase in the signal-to-noise ratio. Moreover, at a certain point, the spatial resolution is also affected (Flenner *et al.*, 2020 ▸; Waske *et al.*, 2010 ▸). In order to analyze these data sets quantitatively, reconstructions usually require capable filtering techniques for the segmentation of structural features and subsequent analysis.

In recent years, numerous powerful filtering techniques have been developed, making it possible to reduce the noise [*e.g.* nonlocal means (NLM) filtering (Liu *et al.*, 2010 ▸; Buades *et al.*, 2005 ▸; Diwakar & Kumar, 2018 ▸)]. However, these filters are not usually free of signal loss, leading to a reduction in spatial resolution and therefore hindering detailed segmentation (Schlüter *et al.*, 2014 ▸). In particular, for very fine features at the limit of the spatial resolution of a wide range of nano­tomography setups (∼100 nm), conventional filters often reach their limits. In addition, most of these filters are optimized to reduce random noise. After tomographic recon­struc­tion, the noise has been projected from Radon space to Euclidian space and is no longer randomly distributed (Diwakar & Kumar, 2018 ▸).

One approach for handling tomographic noise by Bruns *et al.* (2017 ▸) uses an iterative NLM filter. The iterative NLM filter is optimized to handle noise in micro­tomographic reconstructions retrieved from homogeneously illuminated projection images of the samples. For nano­tomography, however, the illumination of the sample is often not homogeneous since illumination optics are used (*e.g.* beam-shaping condenser or capillary optics). In addition, a misaligned phase ring can contribute to con­trast changes over the illuminated field of view. Altogether, this can lead to a very inhomogeneous distribution of noise in the reconstructed nano­tomographic data. The iterative NLM filter, for example, has some issues with the changing noise profile in the plane perpendicular to the tomographic rotation axis (*xy* direction).

Several approaches have been made using machine learning (ML) for denoising tomographic data. Yang *et al.* (2018 ▸) and Pelt & Sethian (2018 ▸) showed that ML can be used to perform fast tomography scans and reduce the noise afterwards. While Pelt & Sethian used a high-quality scan for training, Yang *et al.* trained a single high-quality projection of the specimen to denoise the tomographic scan. These approaches, however, require a certain scanning protocol and cannot be used on data acquired without a high-quality reference. In addition, several ML approaches aim for automated segmentation (Furat *et al.*, 2019 ▸; Ali *et al.*, 2021 ▸) or the reconstruction of limited angle tomography data (Pelt *et al.*, 2018 ▸; Würfl *et al.*, 2018 ▸; Huang *et al.*, 2020 ▸).

In this article, we present an approach based on ML applied to standard synchrotron nano­tomography data. The projections of a TXM scan are split into two independent stacks which are reconstructed separately. The resulting two reconstructed stacks are from the same measurement and contain identical information about the sample, but the noise in the two different reconstructions is uncorrelated. This concept (Noise2Inverse) has very recently been proven mathematically by Hendriksen *et al.* (2020 ▸), where artificial noise was added to micro­tomographic data. Real experimental data, however, often suffer from additional artifacts (*e.g.* ring artifacts or movement artifacts), which can be potentially enhanced by the ML algorithm. Here, we prove that the Noise2Inverse concept can be applied to nano­tomographic scans and can improve the reconstruction quality significantly, enabling image segmentation. In order to show the potential of the presented approach, three different types of biological samples were tested in this study: (i) a very low absorbing specimen, with distinct small and regular features (butterfly scale), (ii) a structurally heterogeneous and more absorbing material with irregular features (bone specimen), and (iii) a highly disordered and structurally complex specimen (mouse kidney) as a region of interest (ROI) scan. These structurally very different types of samples were chosen to demonstrate the performance and efficiency of the presented ML approach in com­parison with other filtering methods.

## 2. Materials and methods

### 2.1. Setup

All tomograms were recorded on the X-ray nano­tom­o­graphy setup at the imaging beamline P05 at PETRA III, DESY, operated by Helmholtz-Zentrum Hereon (Ogurreck *et al.*, 2013 ▸; Greving *et al.*, 2018 ▸; Flenner *et al.*, 2018 ▸). The setup offers high flexibility and high-speed nano­tomography down to 6 s, which is suitable for *in situ* experiments (Flenner *et al.*, 2020 ▸). Both absorption and Zernike phase con­trast can be performed at a spatial resolution below 100 nm in 3D. All scans were performed at an X-ray energy of 11 keV.

**Figure 1:**
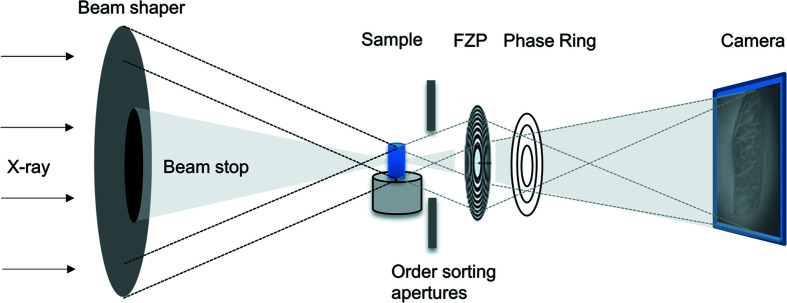
Schematic of a typical transmission X-ray microscopy setup used at P05, PETRA III (DESY, Germany) (adapted from Flenner *et al.*, 2020 ▸).

As shown in the schematic of the setup in Fig. 1 ▸, the incoming X-ray beam is focused using beam-shaping condenser optics (Vogt *et al.*, 2006 ▸; Jefimovs *et al.*, 2008 ▸). Depending on the desired field-of-view (FOV), different sizes of subfields can be chosen. Here, subfield sizes of 50 µm × 50 µm and 100 µm × 100 µm are used. The order sorting apertures block the higher diffraction orders of the beam shaper. A Fresnel zone plate (FZP) produces the image on the detector. The size of the FZP was chosen depending on the desired magnification. A phase ring can be inserted in the back-focal plane to utilize Zernike phase con­trast. Note that Zernike phase con­trast images are non-quantitative. For all experiments, a positive phase con­trast is used, *i.e.* the absorption con­trast is enhanced and not inverted as in the case of negative Zernike phase con­trast. Bright regions therefore represent areas of high electron density and dark areas are characterized by low electron density.

To obtain a reconstruction with high spatial resolution, a sufficient number of projections at equally distributed angles over at least 180° need to be acquired. The amount depends on the sample and also on the setup itself (*e.g.* detector size). Since the stack of projections needs to be divided in order to apply the ML filter approach presented here, the number of projections needs to be doubled. In total, 1760 projections are acquired during a standard tomographic scan of 15 min. The ideal scan time for this setup has been estimated previously (Flenner *et al.*, 2020 ▸).

### 2.2. Samples

The presented types of specimen were chosen to verify the versatility of the presented method: more specifically, the butterfly scales are composed of the biopolymer chitin and the 3D structure gives the scale the bright distinct colour (structural colour). The studied scale is from an Emerald Patched Cattleheart butterfly (*Parides sesostris*) (Starkey & Vukusic, 2013 ▸). Scanning electron microscopy (SEM) images of a similar sample can be found in the supporting information. This sample was measured at an FOV of 50 µm × 50 µm, with a 150 µm diameter FZP and an effective pixel size of 22.2 nm.

Femoral bone was prepared in line with previously published protocols (Stockhausen *et al.*, 2021 ▸). Briefly, femoral cross sections from the mid-diaphysis were cut using a diamond saw and fixed in 3.7% formaldehyde. Thereafter, the sample was embedded undecalcified in glycol methacrylate (Technovit 7200, Heraeus Kulzer GmbH, Wehrheim, Germany) and ground to a thickness of 100 µm using an automatic grinding machine. Images from other high-resolution imaging techniques can be found in Stockhausen *et al.* (2021 ▸). Cylinders of 25 µm diameter were extracted by focused ion beam (FIB) milling and measured at an FOV of 40 µm × 40 µm, with a 130 µm diameter FZP and an effective pixel size of 18.7 nm.

The mouse kidney sample was obtained in the framework of an experiment which was approved by local and national ethics committees, following the European guidelines for the use of animals (APAFIS #8782-201732813328550 v1) (Longo *et al.*, 2020 ▸). The kidney was collected, dehydrated in ethanol and embedded in paraffin for long-term storage. Millimetre-sized samples of the kidney were then cut and glued on the top of pins to perform the X-ray imaging. Histology images of a similar sample are available in the supporting information (Fig. S2). This sample was measured at an FOV of 72 µm × 72 µm, with a 250 µm diameter FZP and an effective pixel size of 35.9 nm.

## 3. The machine learning (ML) denoising approach

Deep learning algorithms are particularly useful for per­forming nonlinear regression. In tomographic denoising tasks, this can be exploited in a variety of ways. One is the Noise2Inverse approach (Hendriksen *et al.*, 2020 ▸), where the image data are split into two independent sets of projection data. A convolutional neural network (CNN) is then trained to project one data set onto the other in the reconstruction domain. As the noise component in the Radon domain is still uncorrelated, only signal information present in both data sets is regressed. In this study, we focus on the practicalities of implementing the Noise2Inverse algorithm for TXM nano­tomography. For further details on the mathematics of the presented method, the reader is referred to Hendriksen *et al.* (2020 ▸).

**Figure 2:**
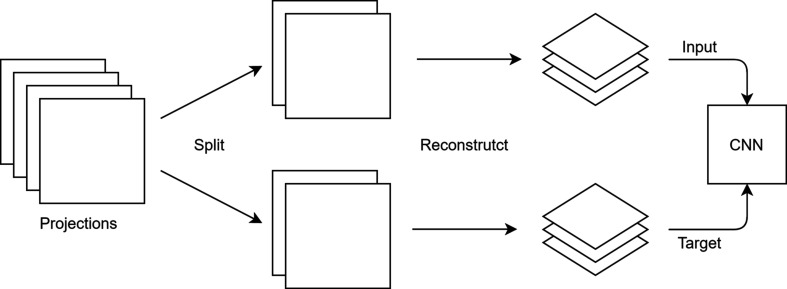
Workflow of the ML denoising approach. The acquired projections are divided into two stacks and reconstructed separately. While one reconstruction is used as an input, the other reconstruction is used as a target. The resulting model can be applied to each of the two reconstructions.

The workflow depicted in Fig. 2 ▸ describes how Noise2Inverse is used to denoise the nano­tomographic scans pre­sented in this article. The workflow is based on an imple­mentation in *Python* using the mixed-scale dense network architecture (MSDNet, https://dmpelt.github.io/msdnet/) (Pelt & Sethian, 2018 ▸; Pelt *et al.*, 2018 ▸). As a first step, the stack of projections is divided into two stacks, each containing every second projection. Each stack of projections is then reconstructed individually. Here we used the GridRec algorithm (Dowd *et al.*, 1999 ▸) with a Shepp–Logan filter implemented in *TomoPy* (Gürsoy *et al.*, 2014 ▸). The resulting reconstructions are used as an input for the ML algorithm, one as the regressor (or input) and the other as the regressand (or target). The training was performed on a Tesla V100 GPU on a stack of 400 neighbouring slices (1024 × 1024 pixels) of the reconstructed stacks. The network was validated and tested on a different stack of 50 slices from the same scan. Training and testing data are not directly neighbouring slices and can therefore differ slightly, making the network more robust and applicable to different samples with a similar structure. Choosing random slices for training and testing instead of neighbouring slices did not show any improvement in performance (see Fig. S3 in the supporting information). Training the network on individual slices (*xy* direction) leads to artifacts in the other direction (*z* direction). Therefore, for each slice, the five closest adjacent slices were used as additional input channels; this eliminates the artifacts and improves the image quality in all directions. The MSDNet model is trained to be applied to a reconstruction of equal noise level, *i.e.* reconstructions with the same number of projections and acquired under the same experimental conditions as the split reconstructions used for training. That means that the trained model only needs to be applied to one of the two reconstructions for obtaining a denoised result, while the other is needed for training only.

**Figure 3:**
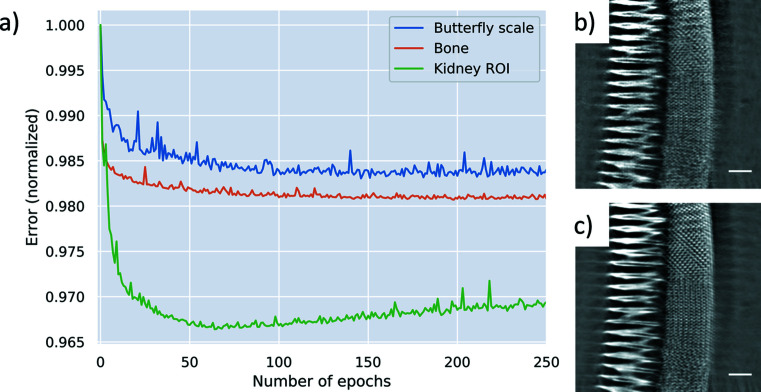
(*a*) The error of the validation set as training progressed. The network applied after (*b*) 5 epochs and (*c*) after 190 epochs. The scale bar is 5 µm.

When applying the algorithm to TXM data acquired at the synchrotron radiation beamline P05 at DESY, we observed that the number of epochs required for training largely depended on the complexity of the sample. As a rule of thumb, the network has to be trained longer when smaller features have to be resolved. While 60 epochs were sufficient for the bone sample to resolve all relevant features (approximately 2 h for 400 slices, 1024 × 1024 pixel), the butterfly scale needed around 190 epochs (>6 h) to resolve the very fine structures (Fig. 3 ▸). As an example, Figs. 3 ▸(*b*) and 3 ▸(*c*) show the improvement of the network from 5 epochs [Fig. 3 ▸(*b*)] to 190 epochs [Fig. 3 ▸(*c*)]. Here, the mean-squared error between the output image and the target images is shown as an indicator for the improvement of the ML network. The increasing error value of the ROI kidney data set points towards overfitting of the network. This is very likely due to the nature of the ROI scans, where features outside the FOV are rotating in and out. Artifacts caused by these features are correlated in both training data sets, but are not necessarily equally distributed in the sample, *e.g.* by changing thickness or composition. Therefore, training of the network was terminated when the error in the validation data set became minimal.

## 4. Results

### 4.1. A high-resolution data set: single butterfly scale

Besides classical neighbourhood filters, such as rank filters like the median filter or convolutional filters like Gaussian and mean filters, there has been much progress in advanced filter techniques that find their application in tomographic de­nois­ing tasks, including anisotropic diffusion, mean shift and non­local means (NLM). The ML-based approach is compared with two 3D filters: a novel variation of the NLM filter, spe­cifically designed for microtomography applications (Bruns *et al.*, 2017 ▸), and a standard 3D median filter with a radius of 2 voxels which was applied after the reconstruction on the 3D stack. For a com­parison of performance, the full stack of projections was also reconstructed. The con­trast-to-noise ratio (CNR) was calculated for all four options using the following formula (Muhogora *et al.*, 2008 ▸): 

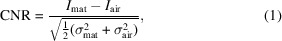

where *I*_mat_ and *I*_air_ are the mean gray values in the material and air, and σ_mat_ and σ_air_ are the standard deviations for these materials.

**Figure 4:**
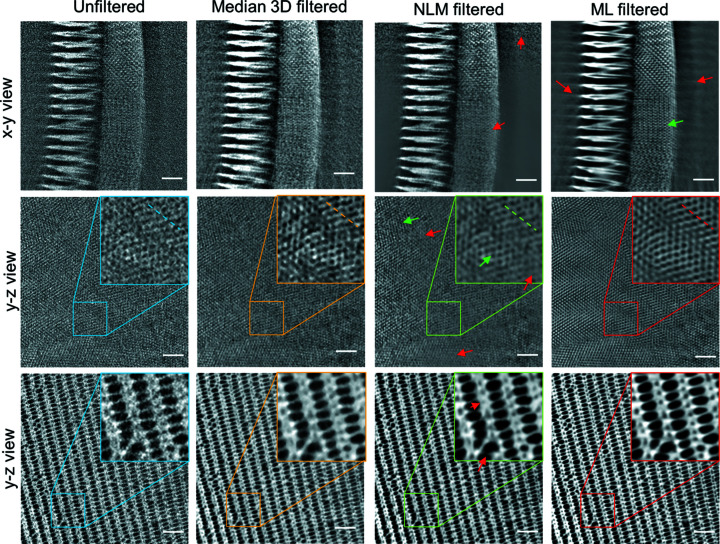
Comparison of the original data set (left) and three filtered data sets of the butterfly scale in an *xy* view and two different *yz* views. The filtered data sets are median 3D filtered (2nd column), iterative nonlocal means (NLM) filtered (3rd column) and ML-based filtered (4th column). The scale bars are 2 µm.

A butterfly scale is a very low absorbing specimen with distinct small and regular features. Therefore, it is the ideal test sample for a high-resolution data set and was measured with a voxel size of 22 nm. Fig. 3 ▸ shows slices and close ups on the tomographic reconstructions in different parts of a single butterfly scale with different denoising filters applied. The noise is dominant with a low CNR (Table 1 ▸) and the nano­structured periodic optical structure is hardly visible in the unfiltered image. It is apparent that straightforward segmentation of this raw data set is likely to fail without any further image processing steps. The median filter reduced the noise and the CNR increases significantly, but also the fine structures are eliminated to a large extent. In particular, the very fine optical nano­structures (*e.g.* the zoomed region in Fig. 4 ▸, 2nd row) close to the resolution limit of the setup often cannot be resolved using conventional filters. Covering only a few pixels in size and therefore comparable to noise in terms of intensity and spatial frequency, the denoising of a butterfly scale is not straightforward. In the ML-filtered images, the periodic structure is clearly visible without any noise (Fig. 4 ▸, 4th column) and the CNR is the highest of all the filters. Since the noise of both stacks is not correlated, the network can learn to distinguish between noise and real structure. This enables the analysis of these very fine-structured biological materials. SEM images of the butterfly scales show the same structural features as observed in the denoised tomography data (see Fig. S1 in the supporting information), proving that the ML data are close to the correct ground truth. The noise reduction in the central part of the NLM-filtered image is comparable to the ML approach, with only a slightly lower CNR (Table 1 ▸). Note that the CNR is limited by the natural variation of gray values inside the material, as well as by the background variations, *e.g.* caused by the halo effect (Lovric *et al.*, 2013 ▸). However, the smallest pores are less well resolved than with the ML approach. In the *xy* view, the changing noise profile of the nano­tomographic setup is visible: the noise level increases slightly towards the edges of the illumination, which may cause problems with the noise profiles con­sidered in the iterative NLM implementation (Bruns *et al.*, 2017 ▸). While a *z*-adaptive noise profile (along the rotation axis of the scan) for ROI scans of irregular shaped samples may be con­sidered, the NLM filter cannot handle a spatially varying noise profile in the *xy* plane correctly (as highlighted by the red arrows in Fig. 4 ▸).

**Figure 5:**
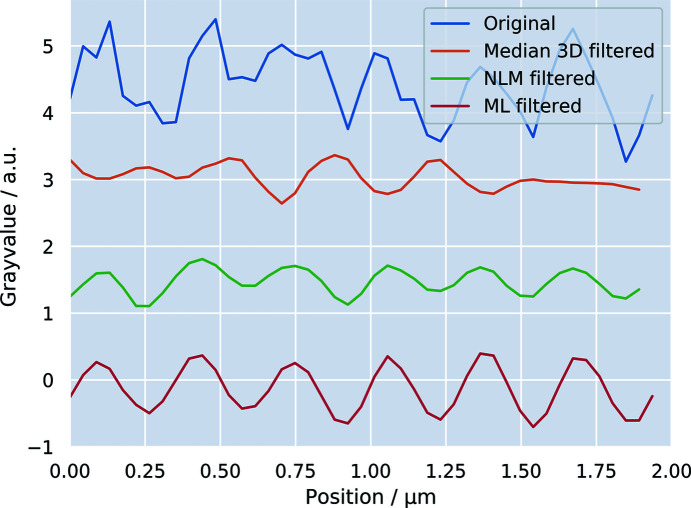
Line profiles along the dashed lines highlighted in the *yz* views found in the 2nd row in Fig. 4 ▸. The filtered profiles were shifted on the ordinate axis for better visibility of the periodic fine structure of the butterfly wing. While the median filter introduces a phase shift to the profile, the NLM filter and ML filter maintain the phase. The amplitude of the signal is best preserved with the ML filter.

The line profiles depicted in Fig. 5 ▸ show that the noise is clearly reduced for both filters. The fitting of a Gaussian pro­file to the peaks of each curve reveals that the ML filter introduces no significant blurring to the data. We found a full width at half-maximum (FWHM) of 200 ± 60 nm for the original data set and the ML filter yields the smallest loss in effective spatial resolution, with an FWHM of 240 ± 30 nm. In com­parison, the peaks in the NLM filter suggest a broader FWHM of 310 ± 80 nm. Looking at the line profile of the median filter, one can recognize that the periodic oscillation does not follow that observed for the raw data and the other two filters, but introduces a phase shift. In this case, the median 3D filter is not well suited for denoising the data as the structures are no longer resolved.

**Figure 6:**
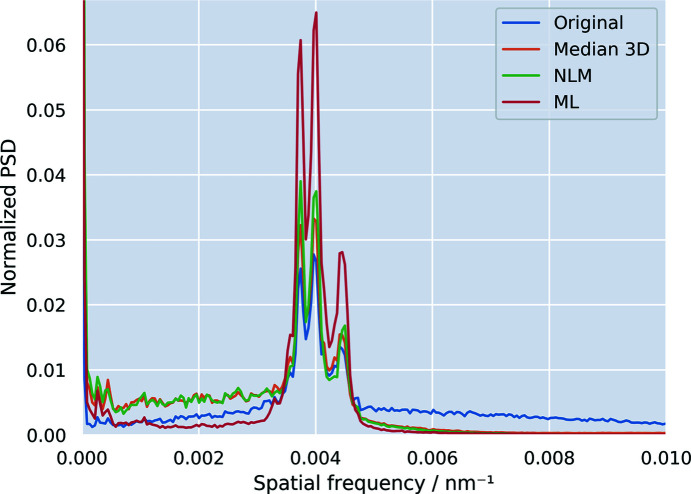
1D power spectral density (PSD) graph of the region of interest in Fig. 4 ▸ (2nd row). The periodic structure of the sample gives rise to frequencies around 0.004 nm^−1^.

In order to visualize the performance of the different filtering methods in a more quantitative way, the 1D power spectrum density (PSD) was calculated *via* Fourier transformation (Fig. 6 ▸) from the fine periodic hole structure in Fig. 4 ▸ (2nd row). The periodic hole structure results in the three main peaks around the spatial frequency of 0.04 nm^−1^ (250 nm) and is well pronounced in the original as well as in the three filtered data sets. The PSD reveals which frequencies are suppressed by the different filters: the NLM and the median 3D filter operate in a local neighbourhood and thus only filter high-frequency noise by design, as shown by the suppression of the higher frequencies in the PSD (Fig. 6 ▸). Lower frequencies remain virtually untouched by both the median and the ML filter. On the con­trary, the ML filter is based on a convolutional neural network and thus allows lower frequencies to be addressed as well. The visual improvements for the ML filter seen in Fig. 4 ▸ that accentuate the details of the butterfly scale are thus explained by an additional removal of low-frequency haze from the image. In con­trast to the NML and 3D median filter, the peak height of the ML-filtered data is not drastically reduced compared to the original data set, impacting the signal-to-noise ratio, as already indicated by the line profiles (Fig. 5 ▸) and CNR values (Table 1 ▸).

Systematic artifacts, such as motion artifacts, streaking, ringing or halo, are not addressed by the ML filter because they are present in both the input and the target reconstruction. Consequently, artifacts in the filtered reconstruction may become more pronounced, as indicated by red arrows (Fig. 4 ▸, ML filtered, *xy* view). Here the ‘ghosting’ stripes likely result from the well known halo effect caused by the Zernike phase con­trast. Nevertheless, we conclude that the ML approach outperforms the NLM filter in detail preservation, as indicated by the green arrows in Fig. 4 ▸.

### 4.2. Region of interest scans: mouse kidney tissue

**Figure 7:**
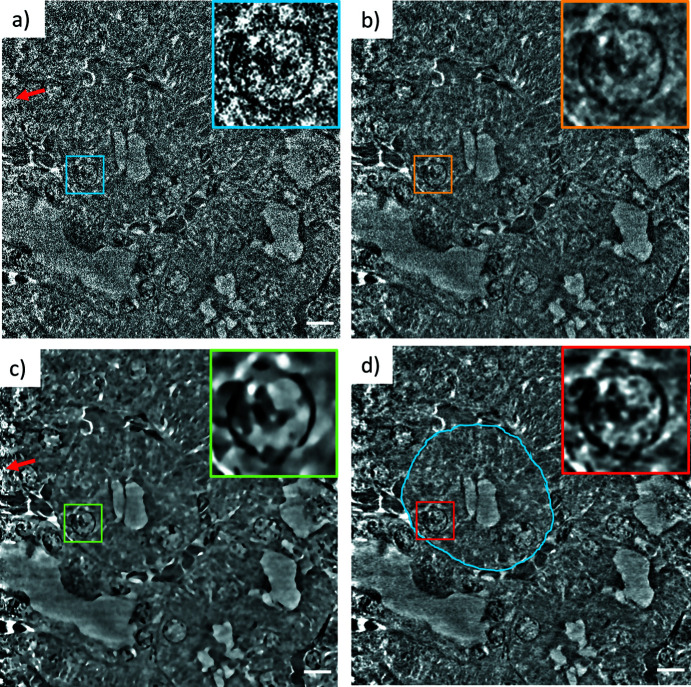
ROI tomography of a mouse kidney in a *yz* view for (*a*) the unfiltered reconstruction, (*b*) the median 3D filter, (*c*) the NLM filter and (*d*) ML applied. The insets show the magnified nucleus of a cell. The blue outline shows a kidney tubular structure. Noise increases at the edges of the image, as indicated by the red arrows. The scale bars are 5 µm.

ROI scans are of particular interest for biological or medical specimens. For the preparation of tissue samples, biopsy punches are often used. Ideally, these cylindrical-shaped specimens are not cut any further in order to avoid preparation artifacts and are therefore much larger than the typical FOV of an X-ray microscope (maximum 80 µm × 80 µm) (Longo *et al.*, 2020 ▸). ROI scans, however, imply that more projections are needed, since the size of the entire object has to be con­sidered for the calculation of the number of projections needed, and not only the FOV (Silva *et al.*, 2018 ▸). Otherwise, aliasing artifacts can arise due to structures outside the FOV that are rotating in and out during the scan, disturbing the high frequencies and reducing the resolution. To reduce such aliasing effects, the ROI scan of the millimetre-sized mouse kidney specimen presented in Fig. 7 ▸ was recorded with an increased number of projections, *i.e.* 5000.

The ROI reconstruction of a mouse kidney sample was filtered using the same methods as described above (Fig. 7 ▸). The unfiltered reconstructed slice in Fig. 7 ▸ is dominated by noise, which increases towards the edges of the image, as indicated by the red arrows. In all reconstructions, a kidney tubular structure [blue outline in Fig. 7 ▸(*d*)] and a nucleus can be identified. As shown by Longo and co-workers, these structures can be found in both nano­tomography slices and histology images (Longo *et al.*, 2020 ▸). The ML-filtered slice [Fig. 7 ▸(*d*)] reveals that the smallest features of the nucleus, shown in the inset image, are much better resolved in com­parison to the median filter [Fig. 7 ▸(*b*)] and the NLM filter [Fig. 7 ▸(*c*)]. Although the median filtering reduces the noise significantly and the structures of the nucleus become clearly visible, there is still a high level of noise, which obstructs further automated analysis. Filtering the projections *via* a median filter instead of the reconstruction reduces the noise more efficiently, but also blurs the fine structures (see Fig. S6 in the supporting information). Again, the NLM filter removes the noise very reliably, but it has its difficulties at the edges of the sample due to the altered noise profile. Very fine features, however, are no longer detectable, as seen in the inset of Fig. 7 ▸(*c*). Here, we see one of the biggest advantages of the ML-based denoising approach in practical applications: it is inherently self-adapting to a changing and/or spatially varying noise profile.

### 4.3. Applicability: bone

**Figure 8:**
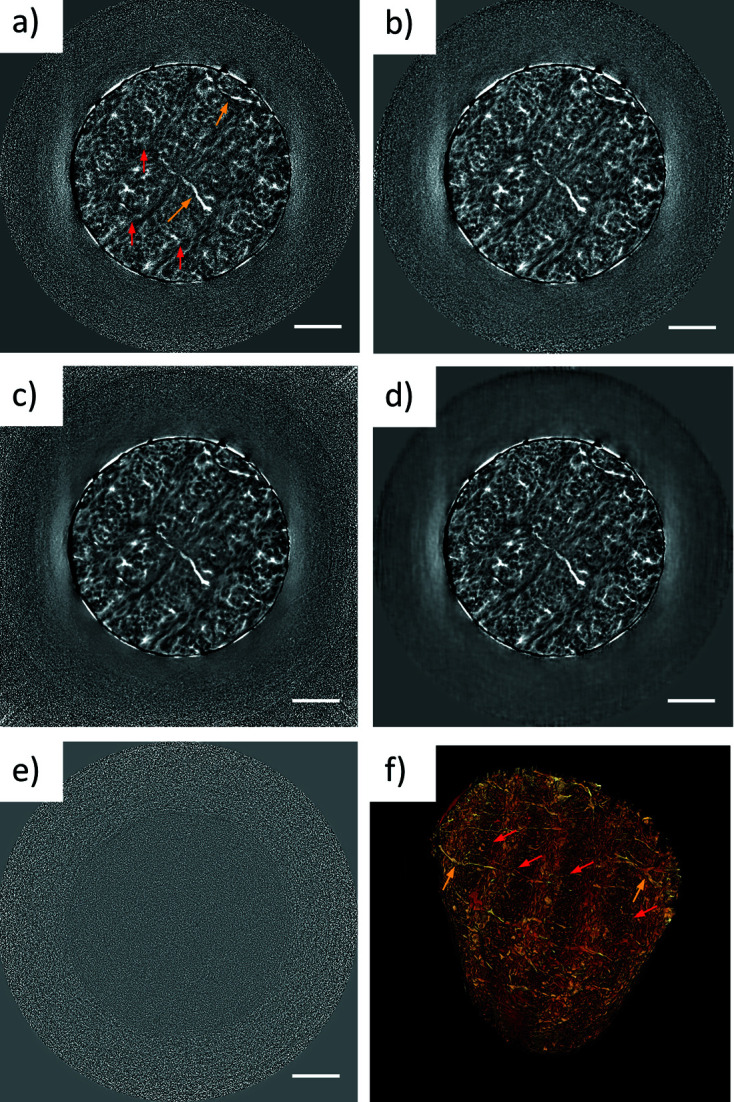
Denoising of a bone sample, showing (*a*) a slice of the unfiltered reconstruction, (*b*) a slice filtered with the median 3D filter and (*c*) the NLM filter. (*d*) The denoised slice performed using a network trained on a different structurally similar sample. (*e*) Difference image of the unfiltered image (*a*) and the ML denoised image (*d*). (*f*) Volume rendering of the bone, achieved by thresholding the ML-denoised tomogram. Red arrows point to the lamellae and yellow arrows indicate the positions of the canaliculi. The scale bars are 5 µm.

A bone sample is displayed in Fig. 8 ▸. Bone is a hierarchically organized and structurally heterogeneous material with characteristic features at each level of hierarchy (Zimmermann *et al.*, 2011 ▸, 2019 ▸). Its matrix is composed of collagen and primarily hydroxyapatite as an inorganic component. The latter defines the absorption properties of the sample, which are much higher than for the butterfly and kidney samples presented above.

The noise level in the unfiltered data [Fig. 8 ▸(*a*)], but also in the median filtered data [Fig. 8 ▸(*b*)], prohibits straightforward intensity-based segmentation and limits the evaluation of any quantitative measure. The iterative NLM filter [Fig. 8 ▸(*c*)] suppresses the noise reliably and reveals all the relevant features, as well as the ML filter, but since the filter is not optimized for the changing noise profile in the *xy* direction, the parameters have to be adapted to filter the relevant area. This can lead to an overestimation of the noise in the centre and may also blur fine features.

Once the network is trained, it is not limited to the data set on which the training was performed, but it can also be used on samples of the same kind with similar inner structures, measured using the same experimental setup and with a comparable noise profile. Compared to the time needed for training a network, the application of the trained network to a similar data set requires only a few minutes of computational time. When the trained network is applied to another scan with similar scan settings, this scan needs to be divided into two stacks in order to match the noise level of the reconstruction on which the network was trained. This basically means that the ML approach presented makes it possible to reduce the scan time for a set of similar specimens by 50%, while at the same time keeping the data high quality. Also, for *in situ* experiments with gradually changing structures, this approach appears highly promising.

The effectiveness of the ML denoising in reducing scan times is outlined in Fig. 8 ▸(*d*). Here a network trained on a 30 min scan of one sample was applied to a reconstruction of a 15 min scan of a structurally similar sample. Taking a difference image of the filtered and the unfiltered image, one can recognize which features have been removed by the filter [Fig. 8 ▸(*e*)]. Ideally, this should only be random noise, otherwise it cannot be excluded that structures are ‘invented’ by the ML filter. In the difference image of the ML-filtered and the nonfiltered images, the noise profile of both scans seems to be slightly different, but no structural features can be recognized [Fig. 8 ▸(*f*)], verifying that the ML filter does not invent any structures. A com­parison of the results for two differently trained networks (self-trained and trained on a similar sample) can be found in the supporting information (Fig. S6).

Using nano­tomography, structural variations at the nano­scale can be visualized. The ML-filtered reconstruction was used to enable segmentation and render the bone sample in 3D [Fig. 8 ▸(*f*)]. The nano­tomographic scans thereby helped in revealing nanometre-thick tunnels (canaliculi, yellow arrows) that connect mechanosensitive cells within the bone matrix and play a key role in maintaining bone health (Milovanovic *et al.*, 2013 ▸). Moreover, lamellar structures with alternating collagen fiber orientation (red arrows) between neighbouring lamellae are visualized (Stockhausen *et al.*, 2021 ▸). These structures can also be identified in SEM images (Stockhausen *et al.*, 2021 ▸), which can be used in a complementary manner to the shown 3D volumes, opening doors for correlative imaging.

## 5. Discussion and conclusion

In this article, the application of a novel machine-learning-based denoising method was demonstrated on experimental high-resolution nano­tomography data. The ML filter was compared to a standard 3D median filter and an iterative non-local-means (NLM) filter optimized for synchrotron radiation microtomography data. Both the ML and the iterative NLM filters are able to remove the noise to an imperceptible level. The 3D median filter does not provide acceptable noise removal but smears out the structural features more visibly. Alternatively, using a median filter on the projections prior to reconstruction minimizes this effect, but the quality of the other two filtering methods is still not reached. The performance of the ML-filtering technique is better or equal to the optimized iterative NLM filter in all the cases presented. The ML filter is the only filter that is able to resolve the fine periodic nano­structures in the butterfly scale. Fine structures down to only very few pixels can be resolved and importantly no blurring is introduced. The advantage of the ML-based filter technique is clearly that the denoising is independent of the noise profiles of the data, *e.g.* noise caused by a misaligned phase ring or suboptimal illumination. On the con­trary, the NLM filter estimates the noise profile for each sample automatically and is *z*-adaptive (or can be manually adjusted), but cannot deal with a changing noise profile in the *xy* direction. The ML approach can also be used to further reduce the sample scan time, since only half of the projections are needed for applying the trained network. A trained network can also be used to denoise a range of similar specimens imaged with the same noise profile. At beamline P05, ultra-fast scans below 3 min are currently only limited by the camera readout time (33 Hz), which hinders the acquisition of a sufficient number of projections to be divided into two data sets.

Artifacts, like rings, caused by defects in the scintillator, or halo-effects appearing in Zernike phase con­trast, are also enhanced by the ML denoising. Therefore, it is essential to have a clean reconstruction, *i.e.* ring artifacts have to be removed before training. Con­sidering the long computation time (approximately 2–6 h training on a GPU), it is advisable to use the ML approach only on data with a low signal-to-noise ratio at the resolution limit. For data with less noise, high con­trast and more coarse structures, the NLM filter is a good alternative, as the required computation time is con­siderably lower (in the range of a few minutes), while the ML approach is the best possible for fine structures near the resolution limit.

The specific advantage of this self-supervised ‘Noise2Inverse’ approach compared to other ML denoising approaches is that it does not need specific reference data (*e.g.* a high-quality reference scan or high-quality projections). This means that the filter can also be applied retrospectively on previously recorded data where a sufficient number of projections were acquired.

We chose TXM data sets for this study as Zernike phase con­trast is more challenging for filtering approaches than, for example, standard absorption TXM, due to the non-uniform illumination induced by the phase rings, as well as the well-known halo effect often observed in these scans. Of course, the filtering method also works on TXM absorption images and also for nonbiological specimens. In summary, the ML approach proves to be a very powerful tool that out­performs conventional filters by eliminating noise without blurring of relevant structural features, thus enabling efficient quantitative analysis.

## Supplementary Material

Supplementary Figures S1 to S7. DOI: 10.1107/S1600577521011139/tv5025sup1.pdf

## Figures and Tables

**Table 1:** Calculated con­trast-to-noise ratio (CNR) for a region of interest (3rd row in Fig. 4 ▸) in a butterfly scale

Filter	CNR
Unfiltered	2.6
Median 3D filter	3.1
Iterative NLM filter	3.3
ML filter	3.4
