# Supplementary material for: Machine learning denoising of high-resolution X-ray nano­tomography data
Source: J Synchrotron Radiat. 2022 Jan 1;29(Pt 1):230–8. doi: 10.1107/S1600577521011139 (PMC8733986; doi:10.1107/S1600577521011139)
Supplement: Supplementary file 1 [file s-29-00230-sup1.pdf]

JOURNAL OF SYNCHROTRON RADIATION

Volume 29 (2022)

Supporting information for article:

Machine learning denoising of high-resolution X-ray nanotomography data

Silja Flenner, Stefan Bruns, Elena Longo, Andrew J. Parnell, Kilian E. Stockhausen, Martin Müller and Imke Greving

Machine learning denoising of high resolution X-ray nanotomography data: supplemental document

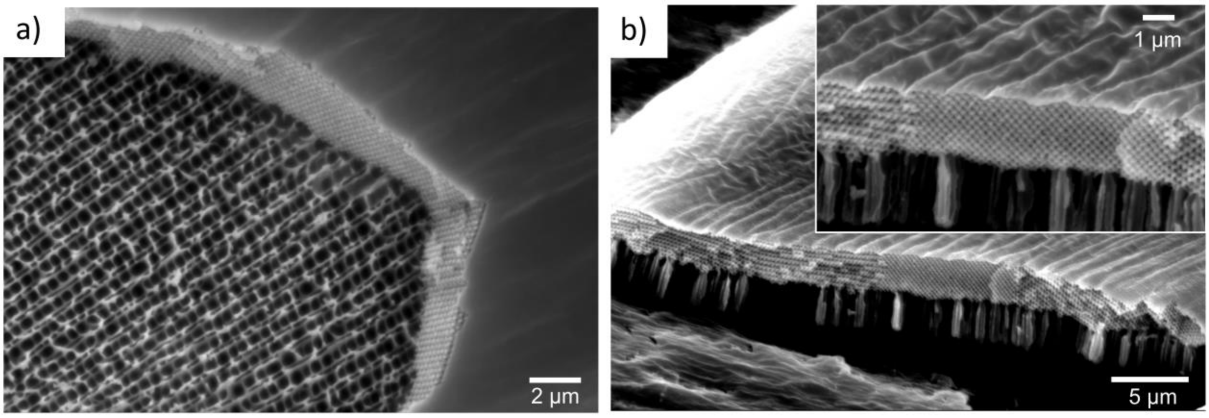

Fig. S1 Scanning electron microscopy (SEM) images of a butterfly scale (Parides sesotris). a) View from an angle similar to figure 4, 3rd row. b) The regular lattice structure and its variation in orientation inside the scale. Inset shows zoomed in region.

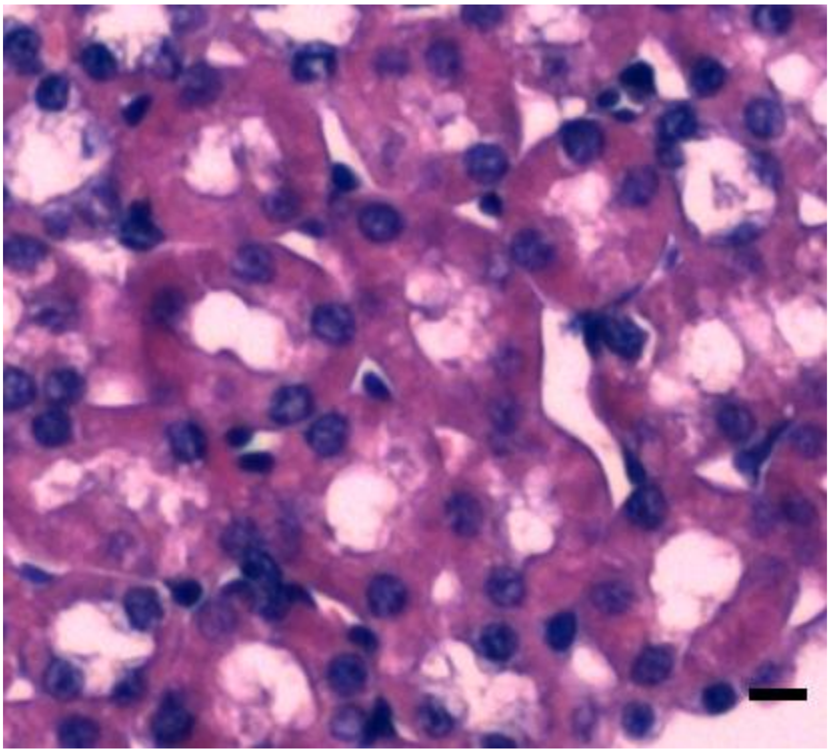

Fig. S2 Histology image of a mouse kidney. Scale bar: 5 µm. (Adapted from Longo *et al.*).

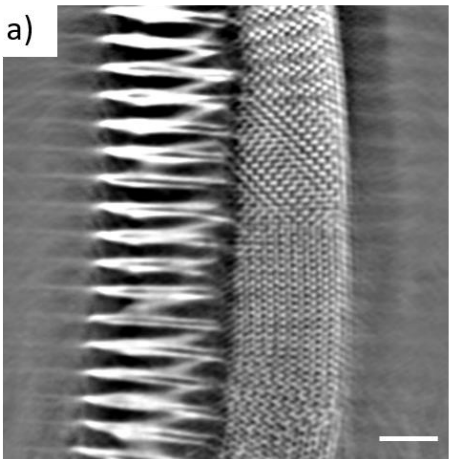

Fig. S3 Results from training using a different selection of training and testing slices. a) Using 400 neighboring training slices and 50 separate slices for validation and testing. b) Using 400 random slices for training and 50 testing inside the sample. No clear differences are visible. Scale bars: 2 µm.

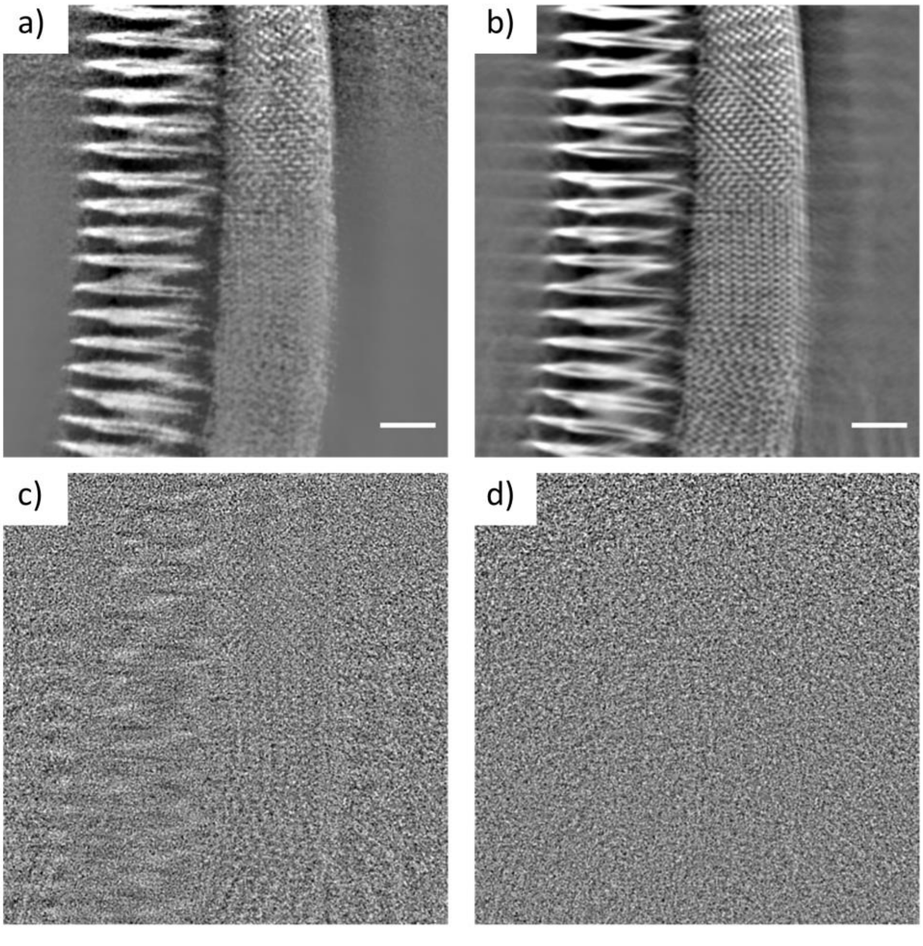

Fig. S4 Difference images. a) NLM filtered, b) ML filtered. c and d show the differences to the unfiltered reconstruction. In c) structures are visible, while in d) only noise is filtered out. Note the changing noise profile from the bottom to the top of the image, which is filtered out by the ML filter, but cannot be removed by the NLM filter. Scale bars: 2 µm.

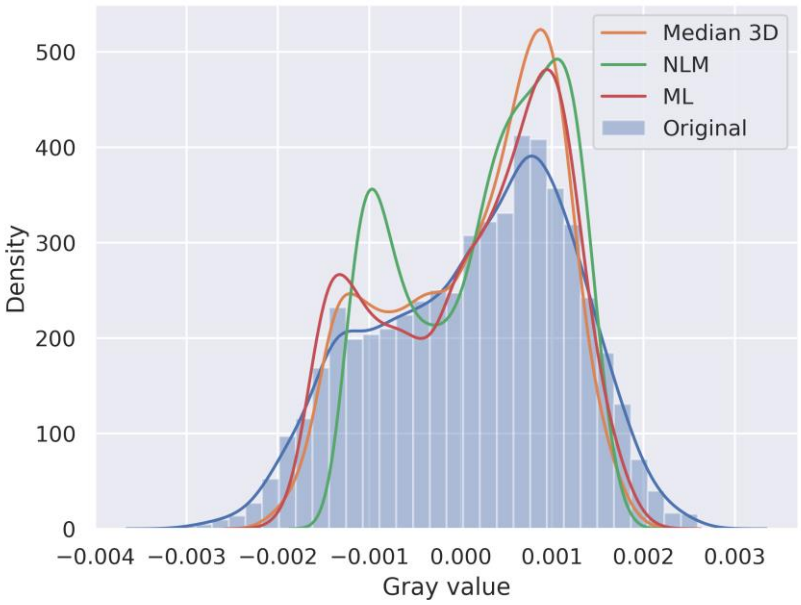

Fig. S5 Histogram of the zoomed ROI in figure 4, third row for the different filters applied. The histogram of the original dataset is shown as light blue bars. The separation of the two peaks (indicating the two main components) is most pronounced in the NLM filtered, followed by the ML filtered. The separation is an indicator of how easy the two components can be segmented via thresholding.

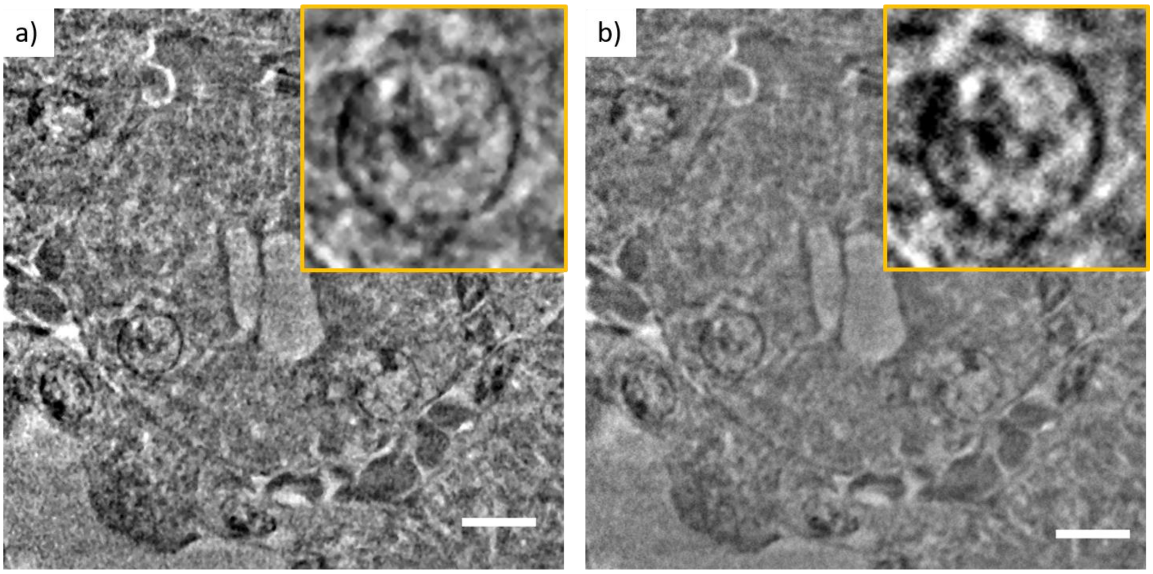

Fig. S6 Comparison of median filters. (a) Median 3D filter applied after the reconstruction and (b) median 2D filter applied prior to the reconstruction. Scale bar: 5 µm.

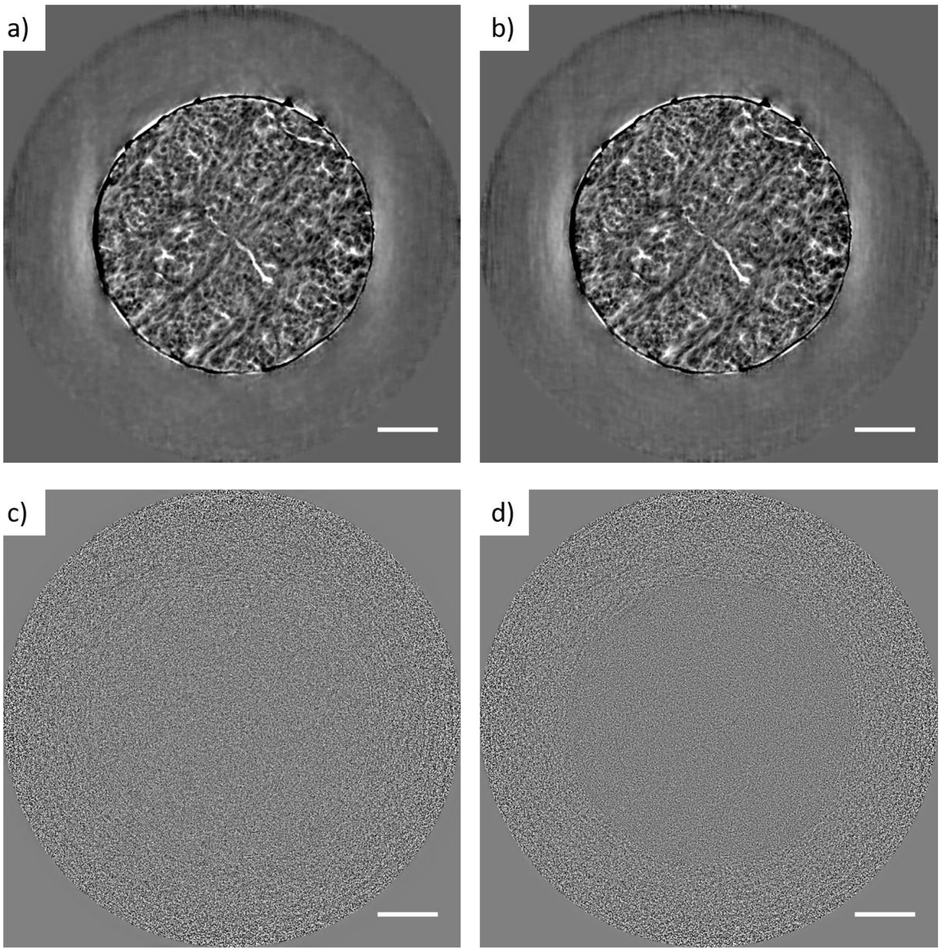

Fig. S7 Comparison of two different trained networks. a) was trained on the data itself, while b) was trained on a scan with twice the exposure time. c) and d) show the differences of the filtered datasets to the original dataset. The slightly different noise profile is visible in the center of d). None of the difference images show any structural features, indicating that the ML network does not invent any structures. Scale bars: 5µm.
